# RSM based optimization of nutritional conditions for cellulase mediated Saccharification by *Bacillus cereus*

**DOI:** 10.1186/s13036-018-0097-4

**Published:** 2018-05-03

**Authors:** Fouzia Tabssum, Muhammad Irfan, Hafiz Abdullah Shakir, Javed Iqbal Qazi

**Affiliations:** 1 Microbial Biotechnology Laboratory, Department of Zoology, University of the Punjab, New Campus, Lahore, Pakistan; 2 Department of Biotechnology, University of Sargodha, University road, Sargodha, Punjab Pakistan

**Keywords:** *Bacillus* sp. 16S rRNA, Cellulase, RSM, *Labeo rohita*

## Abstract

**Background:**

Cellulases are enzyme which have potential applications in various industries. Researchers are looking for potential cellulolytic bacterial strains for industrial exploitation. In this investigation, cellulase production of *Bacillus cereus* was explored while attacking poplar twigs. The bacterium was isolated from the gut of freshwater fish, *Labeo rohita* and identified by 16S rRNA gene sequencing technology. Various nutritional conditions were screened and optimized through response surface methodology. Initially, Plackett-Burman design was used for screening purpose and optimization was conducted through Box-Bhenken design.

**Results:**

The maximum cellulase production occurred at 0.5% yeast extract, 0.09% MgSO_4_, 0.04% peptone, 2% poplar waste biomass, initial medium pH of 9.0, and inoculum size of 2% *v*/v at 37 °C with agitation speed of 120 rpm for 24 h of submerged fermentation. The proposed model for optimization of cellulase production was found highly significant. The indigenously produced cellulase enzyme was employed for saccharification purpose at 50 °C for various time periods. Maximum total sugars of 31.42 mg/ml were released after 6 h of incubation at 50 °C.The efficiency of this enzyme was compared with commercial cellulase enzyme revealing significant findings.

**Conclusion:**

These results suggested potential utilization of this strain in biofuel industry.

## Background

The most abundant and freely available renewable source of energy on earth is cellulose, which could be converted into valuable products such as sugars and biofuels. Conversion of cellulose into valuable products is carried out by various microbes like bacteria and fungi which secrete cellulose degrading enzymes. Complete conversion of cellulose into sugars is done by cellulase enzyme complex. This enzyme complex consists of endoglucanase (EC 3.2.1.4) which acts on internal bonds of cellulose to produce glucan, exoglucanase (EC 3.2.1.91) which acts on ends to produce cellubiose and β-glucosidase (EC 3.2.1.21) which then yields glucose units [1, 2]. The cellulase enzyme complex is produced by a variety of microbes like bacteria, yeast and actinomycetes.

Among various microbes, bacterial strains are widely used for cellulase production due to their fast growth, less energy utilization, easy genetic manipulation and ease of handling [3, 4]. Of the different bacterial genera, *Bacillus* genus is most widely used because it produces alkali-stable and thermostable cellulases [5–9] and other polysaccharide degrading enzyme which are extracellular in nature [10, 11]. Due to these properties of cellulases, researchers are being attracted for their utilization in various industrial sectors like detergent, pulp and paper, wine, brewery, feed and agriculture and in food [5, 12].

Production of these enzymes in labs and industries is carried out by solid state and submerged fermentations [13]. In most of the studies, submerged fermentation process is preferred over solid state fermentation because of ease in performance and efficient heat transfer [14]. For the production of industrial enzymes, cost of growth medium is very important which affects the feasibility of the process. The main factors which affects the growth and product from microbes are carbon source, nitrogen source and other inorganic salts [15, 16]. So, formulation of critical medium components plays a vital role in the production of desired products.

In most of the studies, *rohita* fish gut microflora showed ability to produce variety of enzymes like cellulase, lipase, protease, amylase, chitinase, tannase and phytase because gastrointestinal tract of fish have diverse nutrient flow [17–19]. The cellulolytic potential of many bacteria like *Aeromonas* sp., *Bacillus* sp. has been reported from the gut of *Labeo rohita* [20, 21]. It is very important to explore the gut microflora of fishes which could have association with diverse enzymes production. These bacterial isolates might play an important role in biofuel industry after degradation of cellulose of plant materials.

Various approaches such as one factor at a time (OFAT) and response surface methodology (RSM) have been employed for screening and optimization of various process parameters during fermentation process. In previous reports most of researchers used OFAT, but this approach requires more time and interaction of medium components with each other is also not studied. To overcome this problem, response surface methodology is being widely used because in this we can do more experimental trials within short time with accuracy and each medium component has interaction with each other. Now this time, response surface methodology is more widely used approach for optimization studies in various processes [22–25]. Considering these facts, we made attempt to isolate and identify cellulolytic bacterial strain originally isolated from the gut of *Labeo rohita*, optimize medium through response surface methodology and application of the cellulase enzyme for saccharification process.

## Results and discussion

**Fig. 1:**
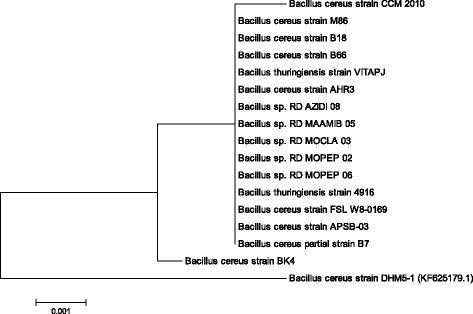
Phylogenetic analysis of *Bacillus cereus* isolated from gut of *Labeo rhoita*

**Fig. 2:**
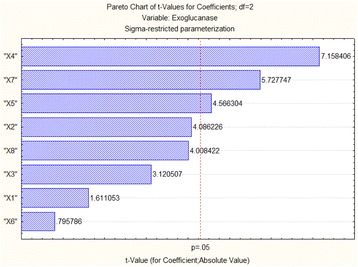
Pareto chart or significant variables for exoglucanase production from *Bacillus* sp

In this study a strain of *Bacillus cereus* was isolated from gut of fish and identified by 16S rRNA gene sequencing technology. BLAST analysis of the sequenced gene revealed 98% homology with *Bacillus cereus* strain as shown in Fig. 1. The isolated strain had potential for cellulase production as confirmed from growth on carboxymethyl cellulose plate stained with Congo red. For cellulase production, various process parameters were optimized and nutritional conditions were screened using placket-Burman design of response surface methodology. The main nutritional components and their levels screened are mentioned in Table 1. Twelve run experiments were conducted for screening of various nutrients for cellulase production and results are mentioned in Table 2. From this experiment the response obtained was analyzed using multiple regression and results showed that three variables i.e. concentrations of yeast extract, MgSO_4_ and peptone were found significant for exoglucanase production as illustrated in pareto chart (Fig. 2).

**Table 1:** Range of parameters used for Placket Burrman design

Parameter	Label	Codes	
		+1	-1
Substrate Conc. (%)	*X*_1_	0.5	5
FeSO_4_.7H_2_O (%)	*X*_2_	0.07	0.13
KH_2_PO_4_ (%)	*X*_3_	0.2	0.6
Yeast extract (%)	*X*_4_	0.15	0.5
MgSO_4_ (%)	*X*_5_	0.06	0.12
CaCl_2_ (%)	*X*_6_	0.025	0.125
Peptone (%)	*X*_7_	0.03	0.05
CoCl_2_ (%)	X_8_	0.05	0.1

**Table 2:** Placket-Burman design for screening of parameters for exoglucanase production in submerged fermentation

Run No.	X_1_	X_2_	X_3_	X_4_	X_5_	X_6_	X_7_	X_8_	Exoglucanase activity (IU)		Residues
									Observed	Predicted	
1	5.0	0.13	0.6	0.15	0.12	0.125	0.05	0.1	0.077	0.069	0.007
2	0.5	0.13	0.2	0.15	0.12	0.125	0.03	0.1	0.09	0.110	-0.020
3	0.5	0.07	0.6	0.5	0.12	0.125	0.05	0.05	0.493	0.462	0.030
4	5.0	0.07	0.2	0.15	0.06	0.125	0.05	0.05	0.295	0.321	-0.026
5	0.5	0.13	0.2	0.5	0.12	0.025	0.05	0.05	0.299	0.329	-0.030
6	0.5	0.07	0.6	0.5	0.06	0.125	0.03	0.1	0.694	0.712	-0.018
7	0.5	0.07	0.2	0.15	0.06	0.025	0.05	0.1	0.161	0.122	0.038
8	5.0	0.07	0.2	0.5	0.12	0.025	0.03	0.1	0.516	0.503	0.012
9	5.0	0.5	0.2	0.5	0.06	0.125	0.03	0.05	0.584	0.557	0.026
10	5.0	0.5	0.6	0.5	0.06	0.025	0.05	0.1	0.297	0.317	-0.020
11	0.5	0.5	0.6	0.15	0.06	0.025	0.03	0.05	0.395	0.395	0.000
12	5.0	0.13	0.6	0.15	0.12	0.125	0.05	0.1	0.077	0.069	0.007

To optimize the concentrations of yeast extract, MgSO_4_ and peptone, Box-Behnken design of response surface methodology with three levels was employed and results are mentioned in Table 3. The response obtained was calculated by second order polynomial regression equation (Eqs.4 and 5).The results showed that maximum cellulase production was observed at concentrations of 0.5% yeast extract, 0.09% MgSO_4_ and 0.03% peptone using poplar biomass as carbon source. The predicted cellulase production under these conditions were almost near to the observed value depicting the accuracy of the model.

4 $$ {\displaystyle \begin{array}{l}\mathrm{Y}\ \left(\mathrm{Exoglucanase}\ \mathrm{activity},\mathrm{IU}\right)=\hbox{-} 9.424+22.851\ {\mathrm{X}}_4+104.91\ {\mathrm{X}}_5+89.52\ {\mathrm{X}}_7\hbox{--} 18.484\ {{\mathrm{X}}_4}^2\hbox{-} 420.65\ {{\mathrm{X}}_5}^2+\\ {}1619.2\ {{\mathrm{X}}_7}^2+57.71\ {\mathrm{X}}_4{\mathrm{X}}_5\hbox{-} 332.86\ {\mathrm{X}}_4{\mathrm{X}}_7\hbox{-} 1100.0\ {\mathrm{X}}_5{\mathrm{X}}_7\end{array}} $$

5 $$ {\displaystyle \begin{array}{l}\mathrm{Y}\ \left(\mathrm{Endoglucanase}\ \mathrm{activity},\mathrm{IU}\right)=\hbox{-} 5.931+23.562\ {\mathrm{X}}_4+76.79\ {\mathrm{X}}_5\hbox{-} 42.6\ {\mathrm{X}}_7\hbox{-} 22.990\ {{\mathrm{X}}_4}^2\hbox{-} 287.6\ {{\mathrm{X}}_5}^2+\\ {}3222\ {{\mathrm{X}}_7}^2+78.33\ {\mathrm{X}}_4{\mathrm{X}}_5\hbox{-} 322.7\ {\mathrm{X}}_4{\mathrm{X}}_7\hbox{-} 1050.0{\mathrm{X}}_5{\mathrm{X}}_7\end{array}} $$

**Table 3:** Box-Bhenken design for exoglucanase production

Run No.	X4	X5	X7	Exoglucanase activity			Endoglucanase activity		
				observed	predicted	residual	observed	predicted	residual
1	0.5	0.12	0.04	1.60	1.60	-0.00	1.80	1.83	-0.03
2	0.325	0.06	0.03	0.860	0.872	-0.012	0.950	0.983	-0.033
3	0.15	0.09	0.03	0.080	0.076	0.004	0.060	0.059	0.001
4	0.15	0.06	0.04	0.430	0.421	0.008	0.390	0.357	0.032
5	0.15	0.12	0.04	0.048	0.052	-0.004	0.015	0.043	-0.028
6	0.5	0.09	0.05	1.260	1.264	-0.004	1.340	1.340	-0.000
7	0.325	0.12	0.05	1.360	1.347	0.012	1.840	1.807	0.033
8	0.325	0.09	0.04	1.660	1.656	0.003	1.640	1.646	-0.006
9	0.5	0.09	0.03	2.200	2.191	0.008	2.159	2.154	0.004
10	0.325	0.09	0.04	1.660	1.656	0.003	1.650	1.646	0.003
11	0.325	0.12	0.03	1.770	1.769	0.0002	2.150	2.121	0.028
12	0.15	0.09	0.05	1.470	1.478	-0.008	1.500	1.504	-0.004
13	0.325	0.09	0.04	1.650	1.656	-0.006	1.650	1.646	0.003
14	0.5	0.06	0.04	0.770	0.765	0.0042	0.530	0.501	0.028
15	0.325	0.06	0.05	1.770	1.770	-0.000	1.900	1.928	-0.028

**Fig. 3:**
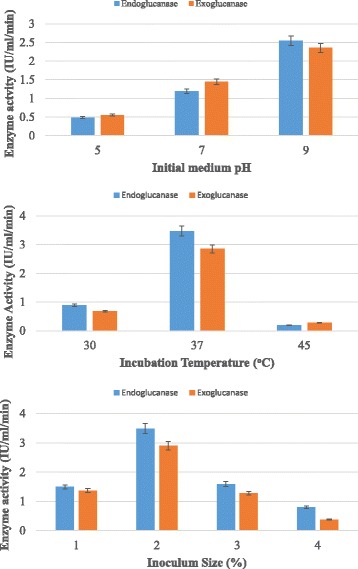
Effect of initial medium pH, temperature and inoculum size on cellulase production by *B.cereus* in submerged fermentation

Various cultural parameters such as medium’s initial pH, inoculum size and incubation temperature were also optimized for maximum exoglucanase production by *Bacillus cereus* in submerged fermentation. Results (Fig. 3) showed that initial medium pH of 9.0 was found most suitable for exoglucanase production in submerged fermentation. Further increase or decrease beyond this level resulted decline in exoglucanase production. *Bacillus cereus* C9 exhibited maximum cellulase production at incubation temperature of 30 °C in submerged fermentation [26]. Mg et al. [27] reported that bacteria isolated from cow dung and municipal solid waste showed optimized production of cellulase at initial medium pH of 6.0 and incubation temperature of 40 °C.In another study [28] an initial medium pH of 6.5 was optimized through central composite design of response surface methodology for cellulase production by *Brevibacillus parabrevis* (MTCC 2208). Previous studies reported that initial medium pH of 7.0–7.2 was most favorable for cellulase production by *Bacillus sp.* in submerged fermentation [29–31].

In the present study, various inoculum sizes such as 1%, 2%, 3%, 4% and 5% *v*/v were tested for maximum production of exoglucanase production by *Bacillus cereus* in submerged fermentation using poplar as substrate. The results (Fig. 3) revealed that inoculum size of 2% (*v*/v) gave maximum titer of exoglucanase production. Similar findings had also been reported by Shankar and Isaiarasu [32] who documented that 2% inoculum size was best for maximum production of cellulase from *Bacillus pumilus* EWBCM1.Whereas Afzal et al. [33] reported that 4% v/v inoculum size was best for maximum production of cellulase by *B. cereus* MRLB1 in submerged fermentation. Ray et al. [34] reported that inoculum size of 3% was best for cellulase production by *Bacillus* sp. *Bacillus subtilis* BY-2 isolated from the gut of the Tibetan pig’s intestine gave maximum yield of cellulose production with inoculum size of 4% [35].

**Fig. 4:**
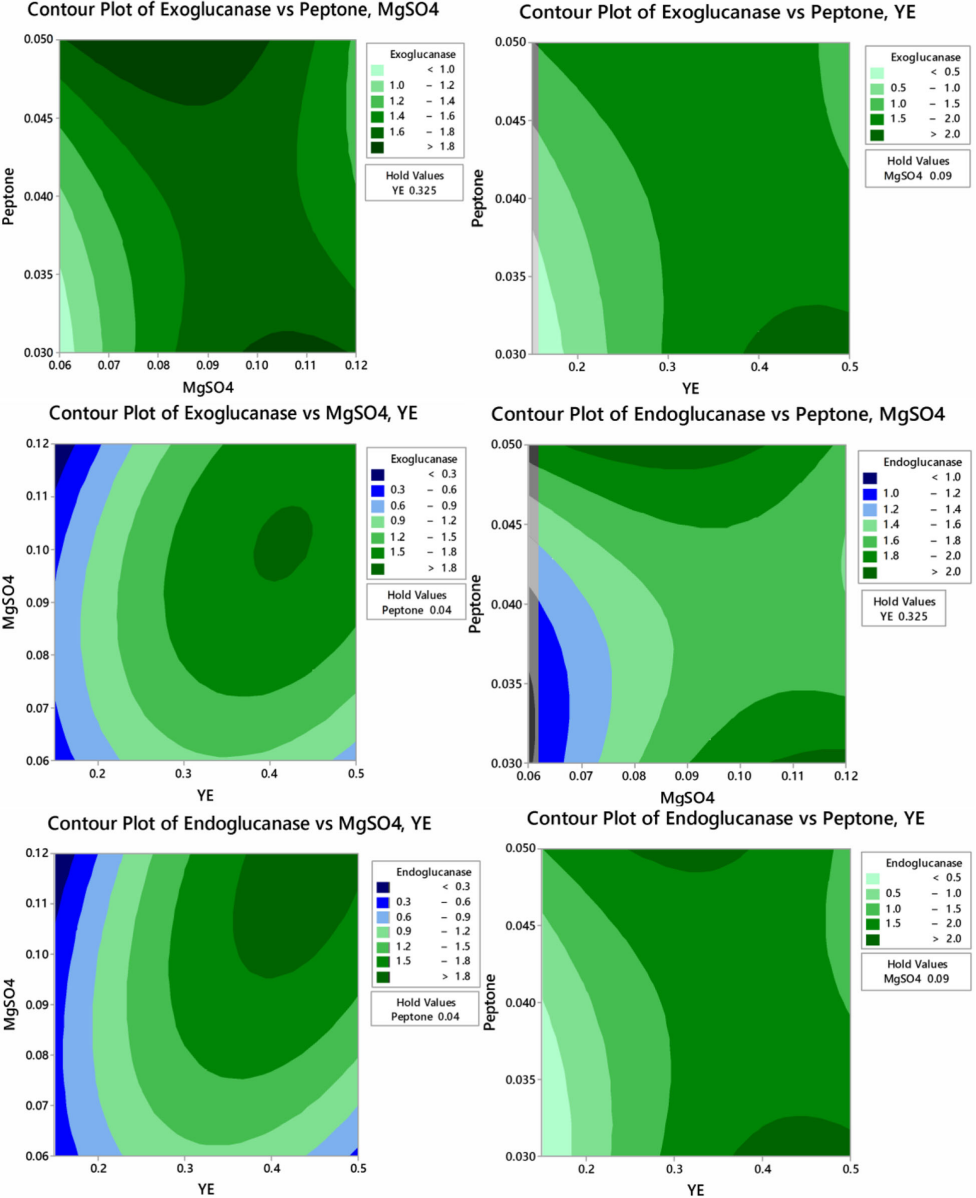
Contour plots of cellulase production from *Bacillus cereus* in submerged fermentation

Figure 4 depicts the contour plots for exoglucanase production by *Bacillus cereus* in submerged fermentation showing interaction of variables. In this investigation peptone, yeast extract and MgSO_4_were found significant for cellulase production in submerged fermentation using *Bacillus cereus.* Sharma et al. [36] optimized various medium components through response surface methodology and reported that peptone (4.94 g/L), ammonium chloride (4.99 g/L), yeast extract (2.00 g/L),Tween-20 (0.53 g/L), calcium chloride (0.20 g/L) and cobalt chloride (0.60 g/L)were significant components for cellulase production using *Bacillus tequilensis* S28 in submerged fermentation. Thakkar and Saraf [37] statistically optimized media for cellulase production and reported that importance of MgSO_4_for maximum cellulase production by *Bacillus amyloliquefaciens* MBAA3. Ali et al. [38] screened various medium components for cellulase production and reported that peptone (0.846 g/L), yeast extract (2.14 g/L), KH_2_PO_4_ (3.05 g/L) and MgSO4.7HO_4_ (0.405 g/L) were significant through response surface methodology using *Cellulomonas fimiNCIM-5015* in submerged fermentation. A previous study also revealed yeast extract as best nitrogen source for cellulase production by *B.cereus* MRLB1 [33]. Peptone and yeast extract had significant effect on cellulase production in submerged fermentation using *Bacillus subtilis* [39]. Ammonium sulphate and ammonium hydrogen carbonate was found best nitrogen sources for cellulase production from *Bacillus licheniformis* APS2 MSU and *Bacillus altitudinis* APSMSU isolated from the gut of fish *Etroplus suratensis* [40]. *Bacillus aquimaris* isolated from the gut of *Labeo rohita* utilized ammonium sulphate as nitrogen source for the production of endoglucanase in submerged fermentation [21]. Yeast extract and (NH_4_)_2_SO_4_ has been reported as a source of nitrogen for exoglucanase by *Aeromonas bestiarum* isolated from the gut of *Labeo rohita* [20].

**Fig. 5:**
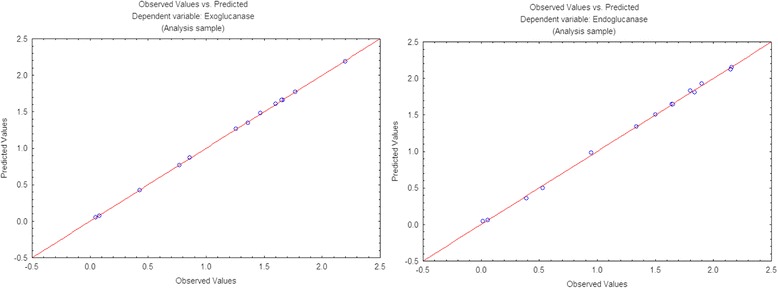
Graph between observed and predicted values

**Fig. 6:**
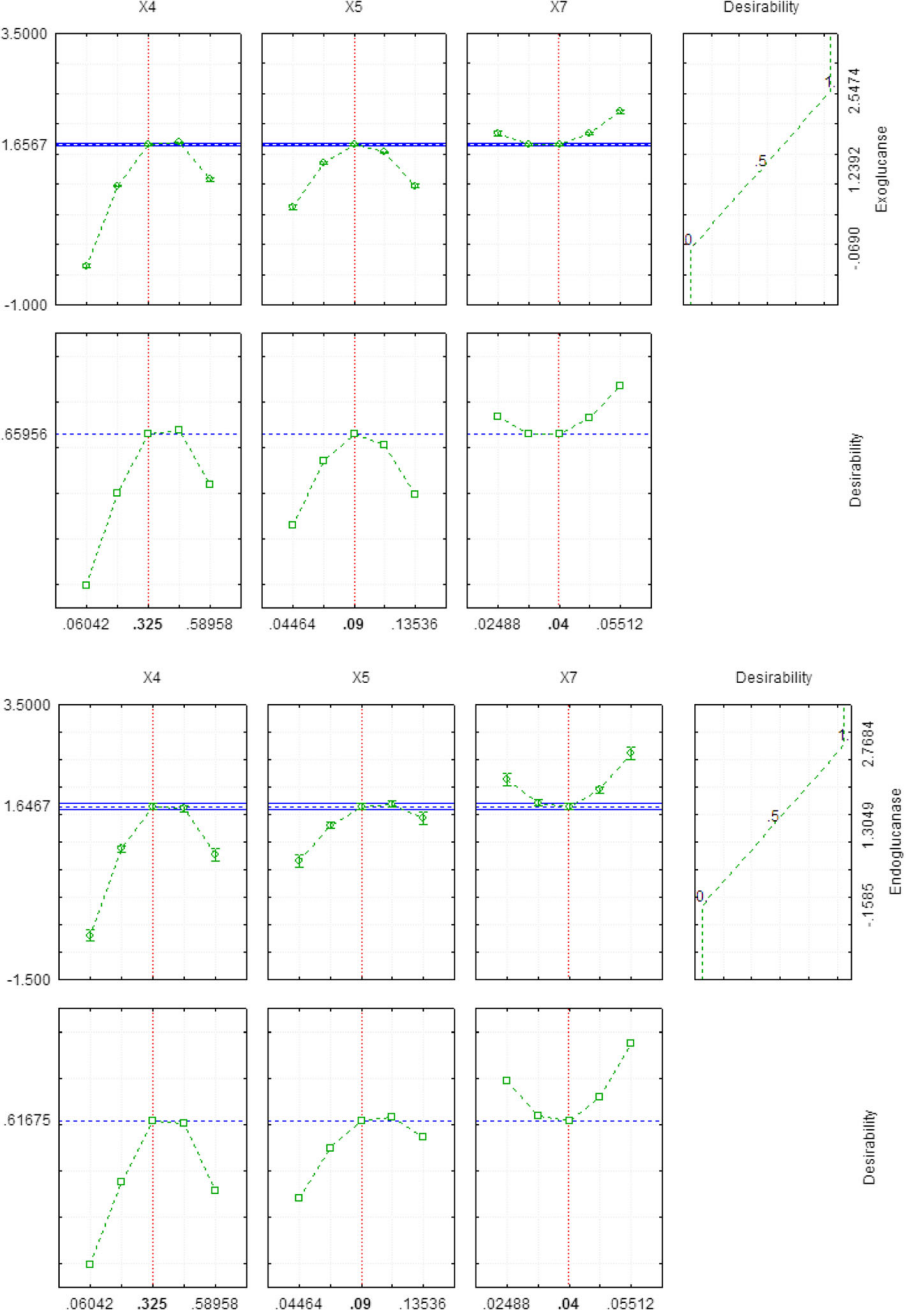
Desirability charts for exoglucanase and endoglucanase production

All the data obtained through experiments were statistically analyzed by analysis of variance. Significant findings were declared on the basis of high Fischer’s test F-value and low probability *P*-value. The proposed model was found highly significant having F-values of4391.8 and 544.58 for exoglucanase and endoglucanase respectively as shown in Table 4. Further significance and accuracy of the model was checked by coefficient of determination (R-value) having value of 99.99% and 99.90% for exoglucanase and endoglucanase, respectively (Fig. 5). The adjusted R^2^ values for exoglucanase and endoglucanase were 99.96% and 99.71%, respectively revealing the goodness of fit of the proposed model. The model results were validated by repeated experimentation as predicted by the optimized values of significant parameters and results obtained were in close agreement with the predicted values of the model (Fig. 6).

**Table 4:** Analysis of variance of cellulase production

Exoglucanase (IU/ml/min)	Sources	DF	Adj SS	Adj MS	F value	*P* value
	Model	9	5.98870	0.66541	4391.18	0.000
	X_4_	1	1.80690	1.80690	11924.11	0.000
	X_5_	1	0.11234	0.11234	741.34	0.000
	X_7_	1	0.11281	0.11281	744.47	0.000
	X_4_^2^	1	1.18320	1.18320	7808.19	0.000
	X_5_^2^	1	0.52920	0.52920	3492.31	0.000
	X_7_^2^	1	0.09680	0.09680	638.81	0.000
	X_4_X_5_	1	0.36724	0.36724	2423.47	0.000
	X_4_X_7_	1	1.35723	1.35723	8956.61	0.000
	X_5_X_7_	1	0.43560	0.43560	2874.62	0.000
	Error	5	0.00076	0.00015		
	Lack of fit	3	0.00069	0.00023	6.91	0.129
	Pure error	2	0.00007	0.00003		
	Total	14	5.98945			
Endoglucanase (IU/ml/min)	Sources	DF	Adj SS	Adj MS	F value	*P* value
	Model	9	7.48850	0.83206	544.58	0.000
	X_4_	1	1.86631	1.86631	1221.50	0.000
	X_5_	1	0.51765	0.51765	338.80	0.000
	X_7_	1	0.19877	0.19877	130.09	0.000
	X_4_^2^	1	1.83040	1.83040	1198.0	0.000
	X_5_^2^	1	0.24737	0.24737	161.90	0.000
	X_7_^2^	1	0.38323	0.38323	250.82	0.000
	X_4_X_5_	1	0.67651	0.67651	442.77	0.000
	X_4_X_7_	1	1.27577	1.27577	834.99	0.000
	X_5_X_7_	1	0.39690	0.39690	259.77	0.000
	Error	5	0.00764	0.00153		
	Lack of fit	3	0.00757	0.00252	75.73	0.013
	Pure error	2	0.00007	0.00003		
	Total	14	7.49614

**Fig. 7:**
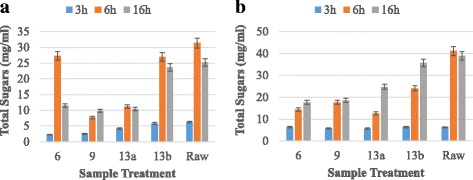
Total sugars produced from hydrolysis of poplar biomass using (**a**) Indigenous enzyme (**b**) Commercial enzyme

**Fig. 8:**
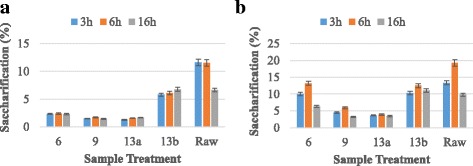
Percent saccharification from poplar biomass using (**a**) Indigenous enzyme (**b**) Commercial enzyme.

The cellulase enzyme produced by this strain was checked for saccharification of pretreated poplar biomass. The saccharification experiments were conducted at 50 °C for various time periods to check the optimum time for maximum sugar production. The efficiency of indigenously produced exoglucanase enzyme was compared with commercial cellulase enzyme. Results (Fig. 7) revealed that indigenously produced and commercial cellulase enzyme yielded maximum release of total sugar of 31.42 mg/ml and 41.18 mg/ml after 6 h of incubation at 50 °C using raw poplar biomass respectively. Maximum reducing sugars produced by commercial and indigenously produced cellulase were 3.85 mg/ml and 2.30 mg/ml after 6 h of incubation at 50 °C respectively. The percent hydrolysis calculated for commercial cellulase and indigenously produced cellulase was 19.25% and 11.50% respectively (Fig. 8). In this whole experiment of saccharification, untreated poplar biomass gave better results as compared to pretreated substrates. The low saccharification rate from pretreated biomass might be due to the production of some inhibitory compounds which might had restricted the enzyme action. These results showed that our in house produced cellulase enzyme gave better results suggesting for its potential utilization in saccharification process.

## Conclusion

These results showed that *B.cereus* (KF625179.1) exhibited cellulolytic potential in submerged fermentation using poplar biomass as substrate. This strain produced maximum cellulase at 0.5% yeast extract, 0.09% MgSO_4_ and 0.03% peptone which was optimized through response surface methodology. The cellulase enzyme effectively hydrolyzed poplar biomass to sugars. These results suggested the potential utilization of this strain in saccharification purposes especially for bioethanol production from plant biomasses.

## Methods

### Isolation and molecular identification of bacterium

The bacterial strain was isolated from gut of *Labeo rohita* [41]. The isolate was identified by 16S rRNA gene sequencing technologyand complete detailed procedure was given in our earlier reports [20, 21]. The sequence obtained was aligned using CLUSTAL W 1.81 [42]. The Phylogenetic tree was constructed by Neighbor-Joining method in MEGA 5.0 (Molecular Evolutionary Genetics Analysis, version 5.0) software [43].

### Fermentation methodology

Fermentation experiments were conducted in 250 ml capacity Erlenmeyer flask and the medium ingredients were used per designed from response surface methodology. The pH of the medium was adjusted to 9.0 with 1 N HCl/NaOH before sterilization. The medium components were sterilized at 121 °C, for 15 min and 15 Psi pressure. After sterilization, 2% *v*/v of the 24 h old vegetative cell culture was raised in nutrient broth transferred aseptically to each of the fermentation flasks. After inoculation, the flasks were incubated at 37 °C with agitation speed of 120 rpm for 24 h. After the termination of the fermentation period, the fermented broth was subjected to centrifugation (Sigma, 2-16PK, Germany) for 10 min at 10,000 rpm and 4 °C for the removal of cell mass and unwanted particles. The clear cell free liquid obtained after centrifugation was used as a crude source of enzyme. Triplicate readings were taken for each of the experiment.

### Analytical methods

Exoglucanase activity was estimated as described in an earlier reports [44]. Reaction mixture containing 0.5 ml of enzyme solution and 0.5 ml of 0.05 M citrate buffer pH 5 containing 50 mg of filter paper was incubated at 50 °C for 30 min. After incubation, 1.5 ml of dinitrosalicylic acid (DNS) solution was added to stop the reaction and test tube was boiled for 10 min in a boiling water bath. Absorbance was taken at 540 nm using spectrophotometer (Spectrophotometer Cecil, CE 2042). One unit (U) of enzyme activity was defined as the quantity of enzyme, which release 1 μmol of glucose under standard assay conditions. Endoglucanase activity was measured using sodium carboxymethyl cellulose (Na-CMC) as substrate. Reaction mixture containing 0.5 ml of crude enzyme and 0.5 ml of 1% CMC (0.05 M citrate buffer, pH 5.0) was incubated at 50 °C for 30 min. After incubation, 1.5 ml of dinitrosalicylic acid (DNS) solution was added to stop the reaction and test tube was boiled for 10 min in a boiling water bath. Absorbance of the color developed was taken at 540 nm using the spectrophotometer (Spectrophotometer Cecil, CE 2042). One unit (U) of enzyme activity was defined as the quantity of enzyme, which released 1 μmol of glucose under standard assay conditions. Total sugars were measured by phenol-sulphuric acid method [45] while reducing sugars equivalent to glucose was measured by 3,5-dinitrosalicyclic acid method [46].

### Enzymatic hydrolysis of substrate

Hydrolysis experiments was conducted in250 ml Erlenmeyer flask using twenty five millilitersof indigenously produced cellulase enzyme having CMCase activity of 2.20 IU/ml/min and FPase activity of 2.159 IU/ml/min. In parallel, commercial cellulase enzyme having FPU of 250 IU/g in citrate buffer pH 5 was used for saccharification at 50 °C for various time periods. The substrate loading of 2% was used for saccharification. After termination of enzymatic hydrolysis the material was centrifuged at 10,000 rpm for 10 min. The supernatant was removed for sugar analysis. Saccharification (%) was calculated using the following formulae as reported in the earlier report [47].

1 $$ \mathrm{Saccharification}\ \left(\%\right)=\frac{\mathrm{Reducing}\ \mathrm{sugars}\ \left(\mathrm{mg}/\mathrm{ml}\right)}{\mathrm{Substrate}\ \mathrm{used}\ \left(\mathrm{mg}/\mathrm{ml}\right)} $$

### Experimental design

Plackett–Burman experimental design was used to screen out and evaluate the relative importance of the medium’s different components as twelve runs experiment for exoglucanase production in submerged fermentation. Each variable was designated and used with a high (+) and a low (−) concentration (Table 1). The nutrient factors tested included concentrations of substrate, MgSO_4_, Yeast Extract, CaCl_2_, CoCl_2_, Peptone, KH_2_PO_4_ and FeSO_4_.7H_2_O. Furthermore, the physical parameters like pH, inoculum size and temperature were optimized through OFAT.

In order to optimize process conditions Box-Behnken design (BBD) was used for cellulase production. The independent variables used were concentrations of yeast extract (X_4_), MgSO_4_ (X_5_) and peptone (X_7_) and their levels are mentioned in Table 3. This design is most suitable for quadratic response surface and generates second order polynomial regression model. The relation between actual and coded values was described by the following equation.

2 $$ {x}_i=\frac{X_i-{X}_{\circ }}{\Delta {X}_i} $$

Where *xi* and *Xi* are the coded and actual values of the independent variable, *Xo* is the actual value of the independent variable at the center point and Δ*Xi* is the change of *Xi.* The response is calculated from the following equation using STATISTICA software (99th edition).

3 $$ y={\beta}_{\circ }+\underset{i=1}{\overset{k}{\Sigma}}+\underset{i=1}{\overset{k}{\Sigma}}\kern0.5em {\beta}_i{X}_i^2+\underset{i}{\Sigma}\underset{j}{\Sigma}{\beta}_{1j}{X}_i{X}_j $$

where Y is the response, k is the number of variables, ß_0_ is the intercept, Xi and Xj are independent variables, *ßi* is the *i*th linear coefficient, ß_ii_ is the *i*th quadratic coefficient and ß_ij_ is the interaction coefficient.

## Abbreviation

BBD: Box Bhenken Design
DNS: Dinitrosalicyclic acid
RSM: Response surface methodology
